# A healthcare claims analysis to identify and characterize patients with suspected X-Linked Myotubular Myopathy (XLMTM) in the Brazilian Healthcare System

**DOI:** 10.1186/s13023-024-03144-7

**Published:** 2024-05-07

**Authors:** Paulo Victor Sgobbi Souza, Tmirah Haselkorn, Jader Baima, Renato Watanabe Oliveira, Fabián Hernández, Marina G. Birck, Marcondes C. França

**Affiliations:** 1 Federal University of Sao Paulo, Neuromuscular Unit, Sao Paulo, Brazil; 2 Astellas Gene Therapies, San Francisco, USA; 3 IQVIA, Sao Paulo, Brazil; 4 IQVIA, Bogota, Colombia; 5 Department of Neurology, University of Campinas (UNICAMP), School of Medical Sciences, Campinas, Brazil

**Keywords:** X-linked myotubular myopathy, XLMTM, Congenital myopathy, Claims analysis, Real-world evidence, DATASUS

## Abstract

**Background:**

X-linked myotubular myopathy (XLMTM) is a rare, life-threatening congenital disease, which is not well-defined. To our knowledge, no studies characterizing the XLMTM disease burden have been conducted in Brazil. We identified and described patients with suspected XLMTM using administrative claims data from the Brazilian public healthcare system.

**Methods:**

Data from 2015 to 2019 were extracted from the DATASUS database. As no XLMTM-specific ICD-10 code was available, a stepwise algorithm was applied to identify patients with suspected XLMTM by selecting male patients with a congenital myopathies code (G71.2), aged < 18 years at index date (first claim of G71.2), with an associated diagnostic procedure (muscle biopsy/genetic test) and without spinal muscular atrophy or Duchenne muscular dystrophy. We attempted to identify patients with suspected severe XLMTM based on use of both respiratory and feeding support, which are nearly universal in the care of XLMTM patients. Analyses were performed for the overall cohort and stratified by age at index date < 5 years old and ≥ 5 years old.

**Results:**

Of 173 patients with suspected XLMTM identified, 39% were < 5 years old at index date. Nearly all (*N* = 166) patients (96%) were diagnosed by muscle biopsy (91% of patients < 5 years old and 99% of patients ≥ 5 years old), six (3.5%) were diagnosed by clinical evaluation (8% of patients < 5 years old and 1% of patients ≥ 5 years old), and one was diagnosed by a genetic test. Most patients lived in Brasilia (*n* = 55), São Paulo (*n* = 33) and Minas Gerais (*n* = 27). More than 85% of patients < 5 years old and approximately 75% of patients ≥ 5 years old had physiotherapy at the index date. In both age groups, nearly 50% of patients required hospitalization at some point and 25% required mobility support. Respiratory and feeding support were required for 3% and 12% of patients, respectively, suggesting that between 5 and 21 patients may have had severe XLMTM.

**Conclusion:**

In this real-world study, genetic testing for XLMTM appears to be underutilized in Brazil and may contribute to underdiagnosis of the disease. Access to diagnosis and care is limited outside of specific regions with specialized clinics and hospitals. Substantial use of healthcare resources included hospitalization, physiotherapy, mobility support, and, to a lesser extent, feeding support and respiratory support.

**Supplementary Information:**

The online version contains supplementary material available at 10.1186/s13023-024-03144-7.

## Background

X-linked myotubular myopathy (XLMTM) is a rare, congenital, centronuclear myopathy with an estimated global incidence of 1 in 50,000 male births [1, 2]. Muscle weakness is the defining characteristic of the disease, which is caused by pathogenic variants in the *MTM1* gene, resulting in lack or dysfunction of the protein myotubularin required for normal development, maturation and maintenance of skeletal muscle cells [3, 4]. The majority of affected individuals are male, though affected females with a milder phenotype of progressive muscle weakness in adulthood have been identified. Approximately 80% of male infants with XLMTM present with the severe (classic) XLMTM form characterized by profound neonatal muscle weakness, hypotonia, and respiratory failure along with an associated early death [2, 5]. For these children, motor milestones are significantly delayed and most fail to achieve independent ambulation [6, 7]. Long-term survivors with the severe phenotype are often non-ambulatory and require ventilator support ≥ 12 h per day [7, 8]. Emerging reports of intrahepatic cholestasis complicating XLMTM suggest a previously unknown tendency toward underlying hepatobiliary disease that is fatal in some cases [9–12]. Among patients in the INCEPTUS natural history study, one quarter had a history of hepatobiliary disease and 91% experienced hepatobiliary disease or hepatic adverse events or laboratory or ultrasonographic abnormalities during prospective monitoring [13]. A minority of patients with intermediate and mild phenotypes can breathe independently for at least a few hours per day and may become ambulatory (often temporarily) and meet some motor milestones [7].

Recent advances in the understanding of XLMTM have led to proof-of-concept and preclinical studies exploring novel therapeutic approaches such as adeno-associated virus (AAV)-mediated gene replacement therapy [14–17], enzyme replacement therapy [18], and PIK3C2B inhibition [19]. However, there are currently no disease-modifying therapies approved for XLMTM and management consists of multidisciplinary supportive care measures that include hospitalization for respiratory illnesses, feeding support, mobility support, physical therapy, orthopedic treatments, and occupational and speech therapy [1, 20]. This extensive healthcare resource utilization, based on United States cost data, imposes substantial clinical and economic burden totaling nearly $900,000 per patient in the first year of life and nearly 1.5 million total for patients who survive the first four years of life [21, 22].

Although there are natural history studies of real-world data from patients living with XLMTM worldwide [5–8, 21], studies of patients living with XLMTM in Brazil are lacking. More data on the disease manifestations and management of Brazilian patients with suspected XLMTM are needed to support development of treatment guidelines (including genetic testing), health care systems assessment, and public policy. This study describes the disease manifestations and medical management of patients with suspected XLMTM using administrative claims data from the Brazilian public healthcare system.

## Materials and methods

### Study design

This retrospective claims database study utilized real-world data from patients with suspected XLMTM treated in the Brazilian Unified Health System (SUS) available at the Brazilian Unified Health System Information Technology Department (DATASUS). The DATASUS is an open national database of anonymized data. Following the Brazilian ethical resolution number 510/2016 [23], ethics committee approval was not required for this analysis.

DATASUS databases used for this study contain administrative claims data, which include inpatient and outpatient procedures and provide information on the procedures performed in the SUS. The administrative data are procedures that use standard code fields from billing records, including demographic information, all procedures prescribed (for ambulatory visits or hospitalizations), number of procedures, severity, in-hospital deaths, length of stay, mean stay, and cost information. All ambulatory or hospitalization procedures available in SUS (approximately 6,500 procedures) follow the standardized procedure list System of Management of the Table of Procedures, Medications, and Orthoses, Prosthetics and Special Materials [24].

The data used in this study was collected from two DATASUS databases: Sistema de Informações Hospitalares [Hospitalization Information System] (SIH) that contains data of hospitalizations (procedures prescribed, information on causes, and costs of healthcare resources utilized) from the public health system [25] representing approximately 75% of Brazil’s population of approximately 200 million people [26] and Sistema de Informações Ambulatoriais [Outpatient Information System] (SIA) that contains data on outpatient visits performed at SUS [27].

Most SIA subsets have an ID code that enables linking the outpatient visits to a single patient. Because SIH does not have patient ID information, a probabilistic record linkage (RLK) was performed to allow longitudinal assessment at the patient level using SIH and SIA, using multiple steps with different combinations of patient information from both databases, such as date of birth, gender and ZIP code. This linkage allows for the opportunity to follow a patient in both systems longitudinally.

We extracted data from January 2015 to December 2019 from SIA and SIH. The date of the first claim for the International Classification of Diseases, 10th revision (ICD-10) code for congenital myopathies (ICD-10 G71.2) for each patient was considered the index date for the analysis. This study period was selected considering that patients with severe XLMTM frequently and rapidly progress to death [21]; hence, a period of approximately 5 years was assumed to be sufficient to understand the natural history of the disease in Brazil. In addition, Brazilian guidelines for managing rare diseases, such as congenital myopathies, were published at the end of 2014 [28]; thus, the 2015 cut-off allows for time to organize the database systems after guideline publication. To avoid any impact of the COVID-19 pandemic on delivery of healthcare services, we did not retrieve data after 2019.

Diagnostic procedures and clinical manifestations for the six most common procedure groups (feeding support, respiratory support, mobility support, physiotherapy, speech therapy and other) were based on procedures codes in the database according to Supplementary Table 1.

### Patient selection

Eligible patients were male, aged < 18 years with at least one claim of G71.2 (congenital myopathy) reported in the database according to ICD-10 and pre-defined diagnostic procedures for genetic testing and/or muscle biopsy. Considering there was no ICD-10 code specific for XLMTM during the time of the study, the code for congenital myopathy was used as a proxy and the cohort defined as patients with suspected XLMTM. In that sense, patients who had at least one claim of G71.2 ICD-10 in the database would be considered as patients with suspected XLMTM for the study, since the disease can rapidly progress to death in many cases, and few claims of healthcare linked to this specific code would be reported.

As an X-linked genetic disorder, XLMTM primarily affects males [18], thus, we only included male patients. Furthermore, we only included patients with evidence of diagnostic tests (i.e., muscle biopsy and/or genetic test) present in the database. However, the results of genetic tests and muscle biopsies are not captured in the database because they are not provided through the Brazilian public healthcare system. These tests are performed at academic medical centers and through biotechnology and pharmaceutical company testing programs. We were also unable to examine perinatal complications common to XLMTM (i.e., decreased fetal movement, polyhydramnios, preterm birth, Caesarean birth, and low one-minute Apgar scores) as inclusion parameters because this information is not captured in the database.

Considering that patients with spinal muscular atrophy (SMA) or muscular dystrophy (such as Duchene muscular dystrophy [DMD]) may present similarly to those of XLMTM, we excluded patients with any claims for these diseases during the study period: ≥1 inpatient or ≥ 2 outpatient claims of either ICD-10 G12.x (SMA) or ICD-10 G71.0 (muscular dystrophy), or ≥ 1 claim of nusinersen dispensation (i.e., SMA treatment).

We attempted to identify patients with “suspected severe” XLMTM by considering their use of both respiratory and feeding support, which are common in the management of XLMTM and less frequently used for other congenital myopathies.

### Outcome

The primary outcomes were age at index date, diagnosis test, race distribution, patient’s residence state and stratification by age at index date (< 5 years old or ≥ 5 years old). Secondary outcomes included the number of patients with XLMTM treated in the public system, follow-up time (in months) stratified by age at index date, and clinical manifestations (claims for physiotherapy, hospitalization care, mobility support, feeding support, speech therapy, respiratory support, and other).

### Statistical analysis

As a descriptive analysis of a secondary database, no statistical hypothesis was tested. Analyses were performed to summarize the data for all patients who met the eligibility criteria in the Brazilian population. Outcomes were summarized as absolute frequencies and percentages (%) for categorical variables and by measures of central tendency (mean or median) and dispersion (standard deviation [SD] or interquartile range [IQR]) for continuous variables. Percentages were calculated using the number of patients with available (i.e., non-missing) data. Analyses was performed for the cohort as a whole and stratified by age at index date (first claim of G71.2) < 5 years old or ≥ 5 years old.

## Results

**Fig. 1:**
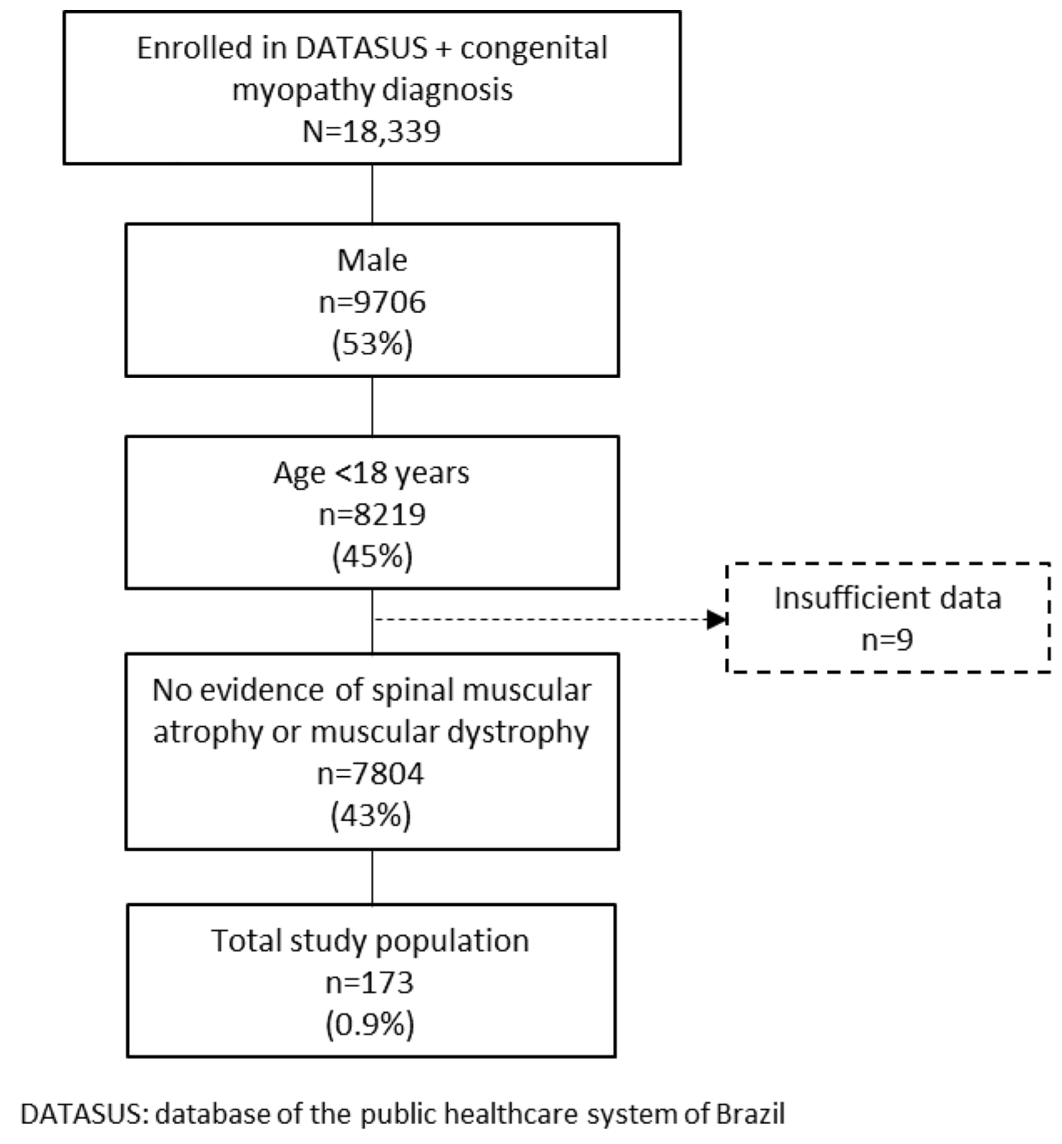
Patient selection

After applying the stepwise algorithm to the initial claims cohort of 18,339 patients, 173 patients were identified as having a congenital myopathy that was suspected to be XLMTM (Fig. 1), of which 64 (37%) were < 5 years old and 109 (63%) were ≥ 5 years old at the index date.

The mean (SD) age at the index date was 2.2 (1.4) years for patients < 5 years old and 10.6 (3.5) years for patients ≥ 5 years old (Table 1). Median (IQR) length of follow-up was 22.5 (10–38) months for patients < 5 years old and 18.0 (8–36) months for patients ≥ 5 years old. Most patients < 5 years old were mixed (36%) or white race (27%); patients ≥ 5 years old had equal representation of white and mixed-race (26% each). Most patients were under the care of a physiotherapist (all ages) or a neurologist (patients ≥ 5 years old) at the index date.

**Table 1:** Patient characteristics

	Age < 5 years old (*n* = 64)	Age ≥ 5 years old (*n* = 109)
Age at index, years		
Mean (SD)	2.17 (1.4)	10.6 (3.5)
Median (IQR)	1.7 (1.1–3.4)	10.5 (8.0-13.1)
Follow-up, months		
Median (IQR)	22.5 (10.0–38.0)	18.0 (8.0–36.0)
Race, n (%)		
Mixed	23 (36.0)	28 (26.0)
White	17 (27.0)	28 (26.0)
Asian	2 (3.0)	5 (5.0)
Black	1 (2.0)	4 (4.0)
Missing	21 (29)	44 (39)
Physician specialty at index, n (%)		
Physiotherapist	41 (85.4)	47 (71.2)
Neurologist	3 (6.3)	16 (24.2)
Medical pathologist/laboratory medicine	3 (6.3)	2 (3.0)
Orthopedist and traumatologist	1 (2.1)	1 (1.5)
Clinical practitioner	0	2 (3.0)
Physician in radiology and imaging diagnosis	0	1 (1.5)

IQR: interquartile range, SD: standard deviation

Nearly all (*N* = 166) patients (96%) were diagnosed by muscle biopsy (91% patients < 5 years old and 99% of patients ≥ 5 years old), six (3.5%) were diagnosed by clinical evaluation (8% of patients < 5 years old and 1% of patients ≥ 5 years old) and one patient was diagnosed by a genetic test.

**Fig. 2:**
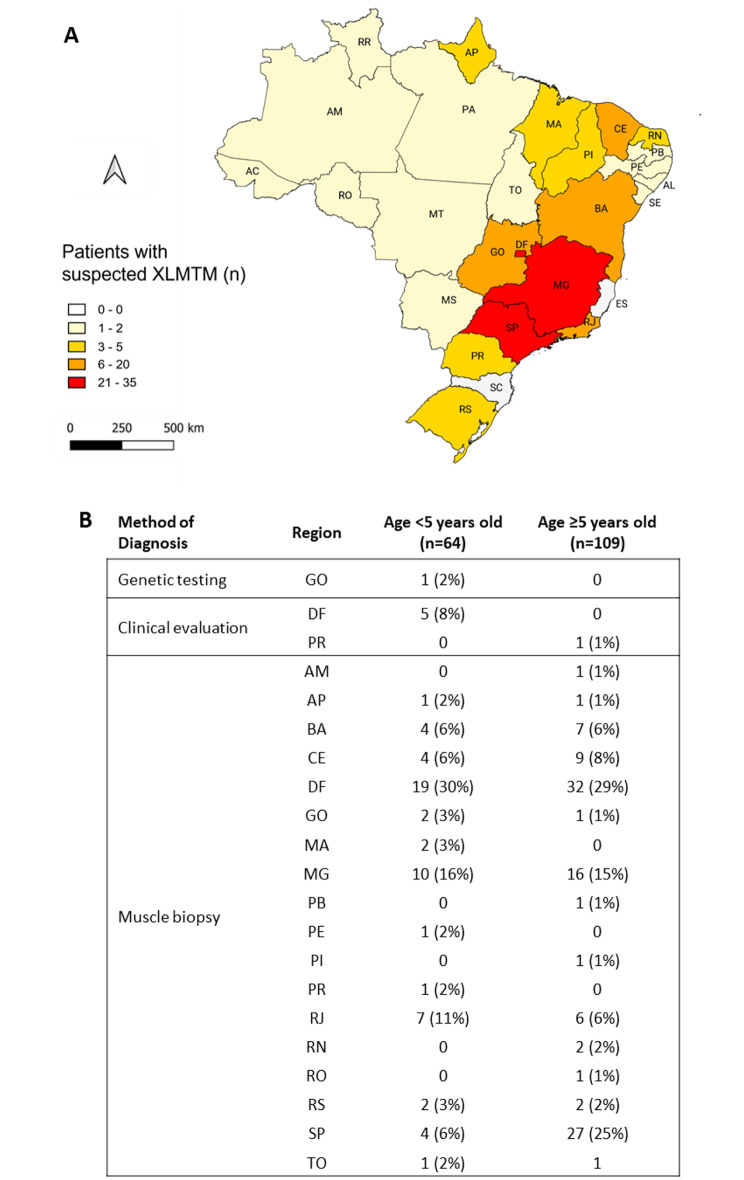
Distribution of patients with suspected XLMTM (**A**) and confirmed XLMTM by genetic test, clinical evaluation or muscle biopsy (**B**) per region in Brazil. AC: Acre, AL: Alagoas, AP: Amapá, AM: Amazonas, BA: Bahia, CE: Ceará, DF: Distrito Federal, ES: Espírito Santo, GO: Goiás, MA: Maranhão, MT: Mato Grosso, MS: Mato Grosso do Sul, MG: Minas Gerais, PA: Pará, PB: Paraíba, PR: Paraná, PE: Pernambuco, PI: Piauí, RJ: Rio de Janeiro, RN: Rio Grande do Norte, RS: Rio Grande do Sul, RO: Rondônia, RR: Roraima, SC: Santa Catarina, SP: São Paulo, SE: Sergipe, TO: Tocantins

The largest proportion of patients resided in the Central-West and Southeast regions of Brazil, of which the most common locales of residence were Brasilia (*n* = 16 [25%] < 5 years old, *n* = 19 [17%] ≥ 5 years old), São Paulo state (*n* = 4 [6%] < 5 years old, *n* = 22 [20%] ≥ 5 years old) and Minas Gerais state (*n* = 10 [16%] < 5 years old, *n* = 20 [18%] ≥ 5 years old) (Fig. 2).

**Fig. 3:**
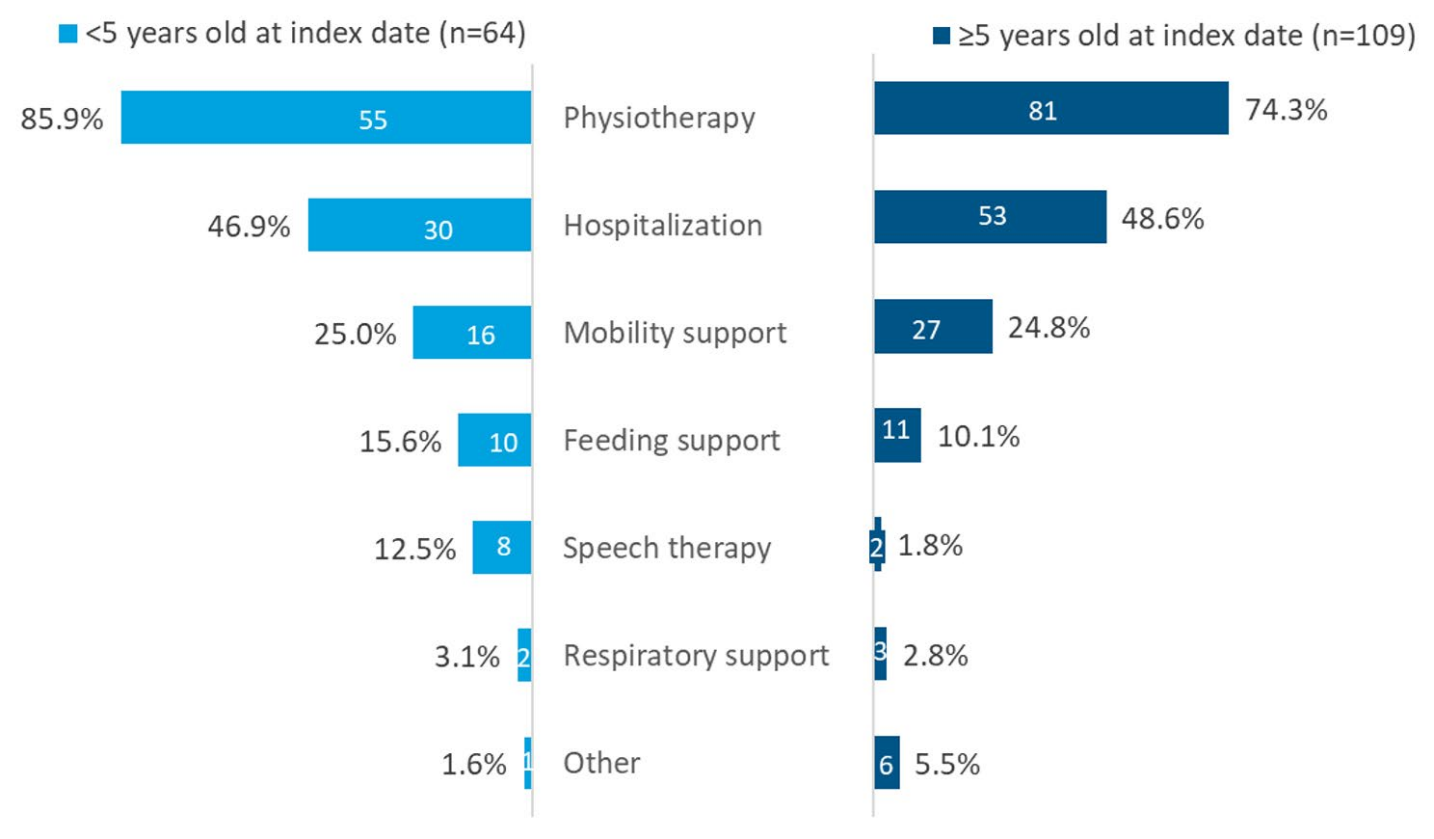
Healthcare resource usage by age at index date

Clinical manifestations and healthcare resource use are shown in Fig. 3. More than 85% of patients < 5 years old and approximately 75% of patients ≥ 5 years old had physiotherapy at the index date. In both age groups, nearly 50% of patients required a hospitalization and 25% required mobility support.

Most patients had healthcare claims after the index date, with a median (IQR) age at last claim of 4.5 (2.9–5.3) years among patients < 5 years old and 12.4 (9.7–15.2) years for those ≥ 5 years old. Less than half of patients had claims for procedures of interest (respiratory support, feeding support, mobility support, physiotherapy, speech therapy, or other related to neuromuscular treatment) before the index date (data no shown).

With regard to identifying a suspected severe XLMTM cohort, respiratory and feeding support after the index date were required for 2.9% and 12.1% of the total study cohort, respectively, suggesting that between 5 and 21 individuals were patients with suspected severe XLMTM; of these, 2 to 10 patients were < 5 years old and 3 to 11 patients were ≥ 5 years old.

## Discussion

The scarcity of accurate epidemiological data on XLMTM in Brazil hinders the estimation of clinical impact and the economic burden of this disease that is needed to drive the decision-making of health system managers.

To the best of our knowledge, this is the first study that attempts to identify and describe disease manifestations and medical management of patients who may have XLMTM in Brazil. Using the DATASUS database, we identified and described the characteristics of 173 patients with suspected XLMTM in the Brazilian healthcare system. Of those, up to 21 patients we considered to have suspected severe XLMTM because of their use of respiratory and feeding support, which natural history studies have shown to be nearly universal in the care of severe XLMTM [5, 21]. It is important to note that a claims analysis of this kind would be more likely to identify patients with a milder phenotype if the most severe patients die at a very early age from lack of access to specialized medical care, while the more severe cases that survive infancy are referred to academic medical centers outside of the public healthcare system. Nonetheless, we did also identify patients who required ventilation and feeding tubes, suggesting a more severe phenotype.

In our DATASUS search 18,339 patients had a congenital myopathy diagnostic code between January 2015 and December 2019, of which 173 (0.9%) were suspected to have XLMTM based on our analysis. Congenital myopathies are a heterogeneous group of orphan diseases diagnosed primarily by muscle biopsy with an estimated pediatric prevalence of 2.73 (95% CI, 1.34–4.12) per 100,000 [29]. Our study is not able to provide an estimate of the incidence or prevalence of XLMTM in Brazil, because the DATASUS database represents claims for care provided within Brazil’s public healthcare system, which serves as the sole healthcare coverage for approximately 75% of Brazilians [30], and not care provided by private healthcare systems. To put our findings in the context of the overall Brazilian population, the nation-wide census conducted in 2019–2020 estimated a total population of 215,786,498 with 2,728,273 live births, of which 1,371,445 were live male births [31]. Our finding of 21 suspected severe XLMTM patients in this population is consistent with another study that used muscle biopsies and genetic test results from two large muscle biopsy banks and a neuromuscular referral clinic in Brazil [32]. Among 3065 biopsies obtained between 2008 and 2013, 63 reported prominent nuclei centralizations, of which 8 were compatible with XLMTM [32]. Based on an estimated 17 million live births in Brazil during that time period [32], those authors calculated a ceiling for estimated XLMTM incidence of 1 in 2,125,000 [32].

Due to the difficulty in defining patients with XLMTM because of the lack of a specific ICD-10 code for this disease at the time of the study, a stepwise algorithm was applied to identify patients with suspected XLMTM. We chose standardized characteristics and criteria regularly used and/or reported in international studies [22]. As a result of this restrictive case selection and the underutilization of genetic testing in Brazil, the number of patients with suspected XLMTM identified in the database may not reflect the true prevalence of XLMTM in Brazil. Among the congenital myopathies in our study, we were able to exclude patients with SMA and muscular dystrophy based on their specific ICD-10 codes; however, we were unable to rule out conditions with XLMTM-like phenotypes, such as congenital myotonic dystrophy type 1 and *ACTA1*-variant congenital muscular dystrophy, due to the lack of specific ICD-10 codes for these diagnoses. Finally, outpatient/inpatient resources are likely to be underestimated due to the intrinsic limitations of the data linking process. Because SIH does not have patient ID information, a probabilistic record linkage was performed to allow longitudinal assessment using SIH and SIA.

Untraceable or unlinked records were removed in order to avoid mismatch or overestimation of the population.

The aim of this study was to identify cases of suspected XLMTM and suspected severe XLMTM in Brazil and attempt to describe their disease manifestations and medical management. Although the small size of the suspected severe XLMTM cohort precludes robust analyses, future studies may include a more in-depth descriptive characterization of these patients.

The estimated median survival of XLMTM patients is 1.8 to 22.8 years and nearly half die by 18 months of age from respiratory failure or related complications, according to data collected from the United States, Europe, and Japan [5, 8, 21]. We stratified our cohort into two subgroups: patients aged < 5 years old or ≥ 5 years old at the index date. Both the international, retrospective RECENSUS study and a recent medical claims analysis show that symptom onset and diagnosis typically occur in the first year of life [5, 22].

We found heterogeneous geographic distribution of patients with suspected XLMTM that may reflect unequal access to healthcare services for neuromuscular disorders, whereby services for diagnosis and management are concentrated in more populous and economically developed regions. These regions are home to centers of excellence for treatment of neuromuscular diseases in the public health care system (i.e., the Sarah Kubitschek Hospitals) or major academic centers, where muscle biopsies and genetic tests are available. This is consistent with findings of Paschoalotto et al. [33] and Horovitz [34] that demonstrated most care centers and services in the area of clinical genetics are concentrated in the most developed regions of the country. We were unable to determine if the database over- or under-represents rural vs. urban regions.

Genetic testing is the standard test to diagnose XLMTM followed by muscle biopsy, and can distinguish this condition from other neuromuscular disorders [3]. Despite publication of the Brazilian guidelines that standardized rare disease management (PCDT) [34], our results show that diagnosis of XLMTM based on genetic testing remains underutilized in Brazil. Nearly all (96%) patients with possible XLMTM were diagnosed by muscle biopsy, and only one patient was diagnosed by a genetic test. This outcome may reflect the difficulty accessing genetic tests in Brazil, as evidenced by a national study showing that only 25–30% of the estimated need in general genetics is being met by experts in the field [34].

With no treatment available to reverse or ameliorate the profound muscle weakness, severe hypotonia, and respiratory distress of XLMTM, disease management aimed at maximizing functional abilities and minimizing medical complications requires intensive use of health care resources [17, 18]. Prior natural history studies have estimated that patients with XLMTM spent 35% of their first year of life in the hospital [5], 87–96% required invasive or noninvasive ventilator support [6, 21], 37% required physical therapy [5], 82% were fed exclusively via feeding tube, and 87% required mobility support [6]. In our study population, 48% of patients with suspected XLMTM had claims for hospitalization, and 79% had claims for physiotherapy. However, only 25% had claims for mobility support, 12% had claims for a feeding tube, and 3% had claims for respiratory support. These contradictory findings may be explained, at least in part by lack of data available in the DATASUS database. Hospitalization is likely underestimated due to intrinsic linkage limitations that require G71.2 ICD-10 code link plus demographic data in the linkage process to provide longitudinal information about the patient. Thus, some healthcare resource data might not be described in the present analysis due to the lack of essential information to link both databases during the linkage process. In addition, most patients receive home ventilatory and feeding support, which are paid for at the state/city level, and thus would not appear in the DATASUS federal database. Due to differing funding across states and municipal levels, especially for home care, federal-level claims data may not reflect the total amount of resources and cost spent on the care of these patients. Moreover, the clinical manifestations of XLMTM during the period leading up to receiving the correct diagnosis are not reflected in our analysis.

## Conclusions

In this real-world study of patients with XLMTM in Brazil, genetic testing appears to be underutilized and may contribute to underdiagnosis of this disease. Regional data suggest there is better access for neuromuscular disease diagnosis and care in specific Brazilian regions where specialized clinics and hospitals are well established. Substantial use of healthcare resources for care of patients with suspected XLMTM included hospitalization, physiotherapy, mobility support, and, to a lesser extent, feeding support and respiratory support.

### Electronic supplementary material

Below is the link to the electronic supplementary material.

Supplementary Material 1

## Data Availability

Researchers may request access to anonymized participant level data, trial level data and protocols from Astellas-sponsored clinical trials at medinfo@audentestx.com. For the Astellas criteria on data sharing see: https://clinicalstudydatarequest.com/Study-Sponsors/Study-Sponsors-Astellas.aspx.
