# Supplementary material for: A healthcare claims analysis to identify and characterize patients with suspected X-Linked Myotubular Myopathy (XLMTM) in the Brazilian Healthcare System
Source: Orphanet J Rare Dis. 2024 May 7;19:188. doi: 10.1186/s13023-024-03144-7 (PMC11077759; doi:10.1186/s13023-024-03144-7)
Supplement: Supplementary file 1 — Supplementary Material 1 [file 13023_2024_3144_MOESM1_ESM.docx]

**Supplementary Material:**

A Healthcare Claims Analysis to Identify and Characterize Patients with Suspected X-Linked Myotubular Myopathy (XLMTM) in the Brazilian Healthcare System

Souza PVS, Haselkorn T, Baima J, de Oliveira RW, Hernández F, Birck MG,
França MC Jr

**Supplementary Table 1. Procedures Code from SIGTAP List**

|  | **ICD-10 code** | **Procedure code – Brazil (SIGTAP list)** | |
| --- | --- | --- | --- |
| Diagnosis procedure |  | *Biopsy* |  |
|  | ― | 0201010283 | Muscle biopsy |
|  |  | 0203020030 | Anatomopathological examination of surgical specimen or biopsy – freezing / paraffin |
|  |  | *Clinical evaluation* |  |
|  | ― | 0301010196 | Clinical evaluation for diagnosis of rare diseases - axis i: 1 - congenital or long manifestation anomalies |
|  |  | *Genetic* *test* |  |
|  | ― | 0202100111 | identification of mutation by amplicon sequencing up to 500 pairs of bases |
|  |  | 0202100065 | DNA analysis by southern blot technique |
|  |  | 0202100073 | DNA analysis by mlpa |
|  |  | 0202100081 | identification of mutation / rearrangements by PCR, sensitive PCR, measurement, qPCR and qPCR sensitive to metilation |
|  |  |  |  |
|  |  | 0202100103 | identification of submicroscopic chromosomic change by array-cgh |
|  |  | 0202100090 | fish in metaphase or interphasic nucleus, for disease |
| Respiratory support | ― | 0301050015 | Follow-up and assessment at home of patients under non-invasive mechanical ventilation (patient/month) |
|  | ― | 0301050066 | Installation or maintenance of home mechanical ventilation |
|  | ― | 0412010127 | Tracheostomy with tracheal or tracheobronchial orthesis |
|  | ― | 0404010377 | Tracheostomy |
|  | ― | 0301100071 | Tracheostomy care |
|  |  | 0301100144 | Oxygen therapy (per day) |
| Feeding support | ― | 0309010039 | Endoscopy gastrostomy |
|  | ― | 0407010211 | Gastrostomy |
|  | ― | 0407010220 | videolaparoscopic gastrostomy |
|  |  | 0309010055 | Enteral nutrition (neonatology) |
|  |  | 0309010063 | Enteral nutrition (pediatry) |
|  |  | 0309010047 | Enteral nutrition (adults) |
|  |  | 0309010071 | Parenteral nutrition (adults) |
|  |  | 0309010080 | Parenteral nutrition (neonatology) |
|  |  | 0309010098 | Parenteral nutrition (pediatry) |
|  |  | 0406020078 | Long-standing catheter |
|  |  | 0309060010 | Double lumen catheter |
|  |  | 0702040150 | Double central venous catheter |
| Physiotherapy | ― | 0302xxxxxx | Physiotherapy |
| Speech therapy | Z50.5 | 030107113 | Individual speech therapy |
| Mobility support | ― | 070101xxxx | Orthesis, prosthesis and special material for locomotion aids |
|  | ― | 070102xxxx | Orthesis, prosthesis and special material - orthopedic |
|  | ― | 070109xxxx | Replacement of orthesis or prothesis |
| Other treatment | ― | 0303040173 | Treatment of muscular dystrophies |
|  | ― | 0303040246 | Treatment of intercurrences of neuromuscular diseases |
